# Integrated Supercritical Fluid Extraction and Pre-Formulation Process of *Punica granatum* L. Pericarp Polar Compounds

**DOI:** 10.3390/molecules28248110

**Published:** 2023-12-15

**Authors:** Sirine Atwi-Ghaddar, Emilie Destandau, Eric Lesellier

**Affiliations:** Institute of Organic and Analytical Chemistry (ICOA), University of Orléans, CNRS UMR 7311, 45100 Orléans, France; sirine.atwi-ghaddar@univ-orleans.fr (S.A.-G.); emilie.destandau@univ-orleans.fr (E.D.)

**Keywords:** eco-extraction, food waste valorization, modified supercritical carbon dioxide, Box–Behnken design (BBD), cosmetic ingredient

## Abstract

Pomegranate (*Punica granatum* L.) is a widely used fruit in the dietary supplement industry due to its richness in bioactive compounds. In this study, an experimental design was applied to optimize supercritical fluid extraction (SFE) of polar compounds of interest (ellagic acid and punicalagins), known for antioxidant and skin care properties from pomegranate’s pericarp. The effects of temperature, modifier percentage, and water additive percentage added in the modifier were explored through a Box–Behnken design, followed by a study of the extraction kinetics. The results indicated that 40 °C, 20% EtOH:H_2_O 80:20 *v:v*, with an extraction duration of 60 min allowed for the highest recovery of the above-mentioned molecules (19.59 mg/g). Due to solubilization issues encountered by the extract, a screening of cosmetic solvents was carried out to solubilize SFE pomegranate extracts and a composition of Gly:H_2_O 80:20 *v:v* was selected. Furthermore, an integrated SFE pre-formulation process of pomegranate pericarp extract (PPE) was elaborated. This allowed for the recovery of the extracts in cosmetic solvent, avoiding a full evaporation. Finally, the stability of the pre-formulated extracts was evaluated and showed high stability for over 3 months at 5 °C.

## 1. Introduction

Pomegranate (*Punica granatum* L.) is an indigenous fruit to a region that spans from what is now modern-day Iran to northern India. It has been grown and appreciated for its fruits in this area for thousands of years. Pomegranates have a long history across multiple cultures including the Phoenicians, Greeks, Arabs, and Romans [1,2].

Over the last ten years, research has focused on the extraction of several parts from the fruit. Preventive and attenuating effects against a variety of chronic and health/life threatening illnesses have been described; including cancer [3], type-2 diabetes [4], and cardiovascular diseases [5]. It is noteworthy to mention that the pomegranate’s edible portion is not the only part containing all the above-mentioned nutraceutical benefits. Research conducted on the non-edible parts of the fruit and tree (such as the seeds, peel, flowers, buds, bark, and leaves) demonstrated that, in some cases, they can contain even higher concentrations of some nutritionally important and biologically active substances coMPared to the edible fruit [6].

Today, the world surface dedicated to the cultivation of pomegranate trees is more than 300,000 ha, and the production seems to be higher than 3,000,000 t. The pomegranate peel or pericarp is considered as a waste product after the juicing process of the fruit. Even though they are still edible, they are typically discarded during juice preparation. Therefore, they present an issue in an industrial setting that is committed to environmental sustainability. This makes them a candidate for waste valorization, especially for the extraction of natural compounds that could later be used as nutraceuticals products, cosmetics ingredients, or as natural preservatives for the food industry [7].

Since the peel and membrane layers of the pomegranate contain more phytochemicals than the edible meat pulp, they are a potential source of bioactive compounds [8,9,10]. According to studies, the peel extracts have strong radical scavenging and antioxidant activity such the DPPH, superoxide ion, hydroxyl, and peroxyl radicals as well as hydrogen peroxide [11,12,13].

Other works have showed that pomegranate pericarp extracts (PPE) contain many polar compounds, like polyphenols such as chlorogenic, caffeic, syringic, sinapic, p-coumaric, ferulic, vanillic, ellagic, gallic, and cinnamic acids [14]. Pomegranate peel cultivar from China was found to have 201.3 and 8.91 mg/g of ellagic acid and gallic acid, respectively [15]. Content in ellagic acid can go up to 50% of the total phenolic compounds depending on the varieties [16]. Additionally, flavonoids like catechin, epicatechin, quercetin, anthocyanins, and procyanidins have been detected in PPE. Flavonoid composition changes as the fruit develops, and the concentration of flavanols and flavones varies between cultivars [17].

This composition may vary according to the freshness of the biomass, as certain studies shows the effect of treatment and packaging of the contents of some flavonoids like rutin. These was identified as the predominant flavonoids in fresh PPE but, due to its high instability during prolonged cold storage (stored for four months by packing fruits inside a polyliner bag), it’s content decreased by 65% [18].

High molecular weight ellagitannins are water soluble compounds that hydrolyze to produce a variety of biologically relevant by-products [19]. Punicalagins (PNGs) are a predominant pomegranate-specific hydrolysable ellagitannins that can transform to punicalin, ellagic, and gallagic acid [19,20,21]; it was demonstrated that PNGs are responsible for more than 50% of the fruit extract antioxidant activity [22]. This bioactivity can be credited to the hydrolysis process of PNGs into ellagic acid (EA) in vivo, and to their ability to cross the mitochondrial membrane in vitro [23,24]. PNGs can be found naturally as two reversible α and β anomers [25,26,27]. Both forms were attributed with antibacterial activity [26,28], with β-anomer representing the major form [27,29].

The extraction of polar compounds from *Punica granatum* L. pericarp have been described in the literature; mostly using traditional maceration extraction and conventional solvents like methanol, ethanol, water, and hydroalcoholic mixtures [30,31,32,33,34]. These techniques have some disadvantages, mainly extraction time that can last days in some cases, and large solvent consumption, that will later require additional evaporation processes. This is why in this study, the use of supercritical fluid extraction has been explored to determine the feasibility and the extraction yield of polar compounds of interest found in PPE, mainly ellagic acid (EA) and punicalagins (PNGs) with both forms PNG_α_ and PNG_β_. In addition, the extraction of natural plant compounds using supercritical fluids is an environmentally friendly approach coMPared to traditional extraction techniques. The supercritical carbon dioxide (SC-CO_2_) is an intermediate state between a gas and a liquid; it exhibits the diffusion, viscosity, and surface tension of a gas as well as the density and solvation capacity of a liquid. Other benefits include the extracts’ spontaneous concentration resulting from the CO_2_’s depressurization at atmospheric pressure. This generates extracts that require minimal refining and post-extraction treatments. Moreover, the use of dimethyl sulfoxide (DMSO) to dissolve PPE was repeatedly mentioned in literature [19,30,32,35,36], in some cases to prevent the precipitation of extracts. Even though DMSO is considered as a non-toxic chemical and has applications in various fields, it is not suitable for cosmetic use. In consequence, PPE requires the use of alternative solvents appropriate for cosmetic formulation.

In this study’s framework, SFE of *Punica granatum* L. pericarp was optimized by the means of experimental design. Ellagic acid and punicalagins were the targeted compounds. A screening of solvents to stabilize the extracts and a development of an integrated SFE pre-formulation process of PPE as a cosmetic ingredient was developed.

## 2. Results

### 2.1. SFE Optimization Using BBD

The SFE of pomegranate pericarp polar compounds was optimized using a BBD. The temperatures 40, 60, and 80 °C were selected due to their ability to influence both the solubility of compounds in the SC-CO_2_ and the diffusivity of compounds in the matrix. The modifier percentage (10, 20, and 30%) and water percentage added as an additive in the modifier (0, 10, and 20%) were chosen for to their capacity to increase the polarity of the extraction phase [37] (Table 1).

The influence of pressure variation on the yield of EA and PNGs from *Punica granatum* L. was not explored in this study and was kept at constant value of 15 MPa. This is based on our previous works treating the extraction of flavonoids from black locust heartwood using supercritical fluid extraction. Indeed, to extract polar compounds, the use of high percentages of modifier and sometimes the use of water as an additive is required in SFE. This may lead to high density of the extraction solvent. In these conditions, the pressure influence on the density of the extraction phase was minimal in the pressure range studied (10 to 20 MPa) [38]. Moreover, the influence of pressure in SFE with pure carbon dioxide have been extensively reported in the literature; it has been noted that extractions performed at high pressures had a higher recovery of volatile fractions and, in some cases, a lower recovery of non-volatile fractions [39,40].

#### Statistical Analysis, Model, and Factor Significance

The determination coefficient (R^2^) for the quadratic model was equal to 0.77 and the adjusted determination coefficient (adj R^2^) was equal to 0.63. This indicated that the model was unable to describe a small part (23%) of the total response variation. The theoretical model demonstrated a satisfactory fit with the experimental data, as seen by the R^2^ value > 0.7, which revealed a good correlation between expected and experimental response values. In addition, the Dixon statistical test was employed and no outliers were identified. This was confirmed by the analysis of residues and the distribution of the measured versus the calculated values. Five repetitions of the central level conditions were performed to evaluate the repeatability of SFE application to the pomegranate pericarp biomass. The relative standard deviation (RSD) of the response was 32.73%, which can be related to the complexity of the matrix.

Interaction factors (X_1_X_2_, X_1_X_3_, and X_2_X_3_) were evaluated during the analysis of the model. They were excluded as they demonstrated signs of data overfitting where the R^2^ increased and adj R^2^ decreased with the addition of each interaction term. Therefore, to avoid this issue, only the quadratic terms were evaluated in this model. The relationship between the yield of total EA and PNGs (Y mg/g) and the three variables (X_1_: temperature, X_2_: modifier %, and X_3_: H_2_O % in modifier) is presented in Equation (1).

Y (mg/g) = 10.14 − 0.6431 × X_1_ + 0.9461 × X_2_ − 0.2988 × X_3_ + 0.005194 × X_1_^2^ − 0.02077 × X_2_^2^ + 0.02927 × X_3_^2^ (1)

The summary of the analysis of variance (ANOVA) for the polynomial model is presented in Table 2. The probability related to F-value and *p*-value were used to determine the significance of each variable. The *p*-value and F-value of the regression model were equal to 0.0097 and 5.4351, respectively, representing a significant regression. The contribution percentage of the equation terms were evaluated to assess the iMPact of each variable on the model. It demonstrated that within the first-degree terms, X_2_ had the highest contribution of 13.6%. This indicated that the modifier percentage is a significant factor of the model. Furthermore, the X_1_ and X_3_ percentage added in the modifier had a contribution of 11.3 and 4.94%, respectively, indicating a non-significant influence on the molecules of interest yield despite the relatively high contribution of temperature. The quadratic terms X_1_^2^ and X_2_^2^ were presented with a contribution of 10.8%. However, X_3_^2^ had the highest contribution with 21.5%, representing a significant effect on the extraction yield of EA and PNGs from *Punica granatum* L. pericarp. This result was also validated by an F-value of 7.9267 and a *p*-value of 0.0183. Consequently, according to the statistical analysis, the most influential factors of the statistical model on the response were the percentage of H_2_O added in the modifier and the modifier total percentage.

The computed optimal conditions that allowed the highest extraction yield of both EA and PNGs were 40 °C, 22.8% of EtOH:H_2_O 80:20 *v:v*, and the predicted extraction yield in these conditions was of 9.232 mg/g. The experiment that corresponded the most to these extraction parameters was experiment n°7 with 40 °C, 20% of EtOH:H_2_O 80:20 *v:v*, with an experimental yield of 11.905 mg/g.

**Figure 1:**
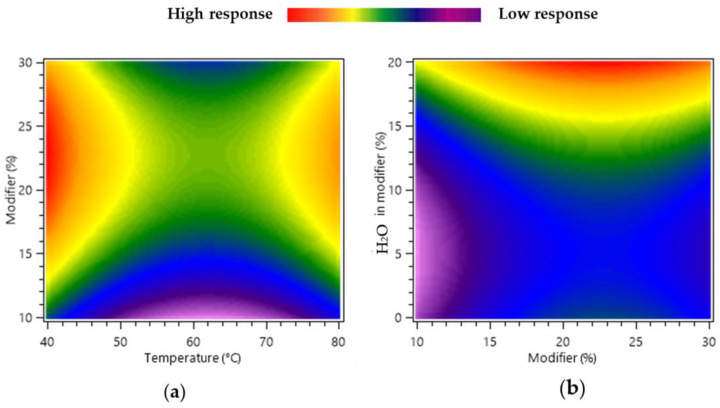
Response surface heat-maps showing the effects of the variation of experimental design factors on the total compounds of interest yield (mg/g) from pomegranate pericarp. (**a**) Interaction between temperature (X_1_) and total modifier percentage (X_2_) and (**b**) interaction between total modifier percentage (X_2_) and H_2_O % in modifier (X_3_).

The variation between the modifier percentage (X_2_) and the temperature (X_1_) is presented in Figure 1a. With X_3_ at central level (EtOH:H_2_O 90:10 *v:v*), the variation of temperatures at 40, 60, and 80 °C resulted in a predicted extraction yield of total compounds of 3.278, 0.805, and 2.486 mg/g. This indicated that the temperature had a limited positive effect on the response with an optimal at 40 °C. The influence of the modifier percentage and H_2_O (%) added as an additive to the modifier on the response is described Figure 1b, at constant temperature (40 °C). The zone with the maximal response was observed when the water percentage was 20% combined with a modifier percentage ranging from 20% to 30%. At a modifier composition of 100% EtOH (without water), increasing the modifier percentage from 10% to 30% slightly raised the yield of total compounds of interest from 0.1095 to 2.415 mg/g. However, using a modifier composition of EtOH:H_2_O 80:20 *v:v* and percentages of 10%, 20%, and 30% of the modifier resulted in yields of 5.842, 9.072, and 8.148 mg/g, respectively. Consequently, a combination of 20% H_2_O and a modifier percentage in the range of 20% to 30% is required to achieve high yields of EA and PNGs from PPE. These variations are related to the solubility change of the polar compounds, and perhaps to the modifications in the matrix solvation, which are favored by both the water and the modifier percentage increase in the examined range.

### 2.2. Validation of Experimental Design and Extraction Kinetics

The results of the BBD indicated that the calculated optimal extraction conditions were 40 °C, 22.8% EtOH:H_2_O 80:20 *v:v* when using an extraction duration of 30 min and a flow rate of 1.5 mL/min. With the aim of assessing the iMPact of the flow rate on the yield of EA and PNGs and determining the extraction time required to exhaust the molecules of interest from *Punica granatum* L., four extraction kinetics were performed with a fraction collection every 15 min for two hours. The modifier composition was EtOH:H_2_O 80:20 *v:v* and a flow rate of 3 mL/min for all extractions. Four experiments were explored in these conditions. The factors investigated were the temperature (40 and 60 °C) and the modifier percentages (20 and 30%). This approach allowed us to investigate finely the influence of temperature (predicted optimal 40 °C) and modifier percentage (predicted optimal 22.8% EtOH:H_2_O 80:20 *v:v*) on the extraction yield with a higher flow rate and longer extraction duration.

**Figure 2:**
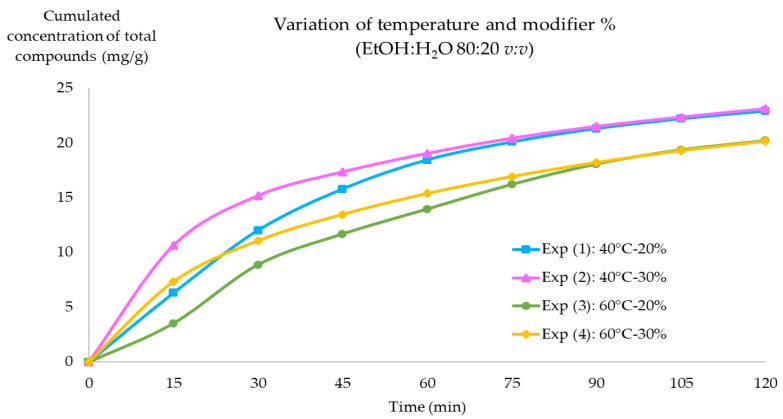
SFE kinetics for the total sum of compounds of interest (EA and PNGs) concentrations in mg/g. Constants: 15 MPa, 1 g of plants, 3 mL/min flow rate, modifier composition EtOH:H_2_O 80:20 *v:v*.

The results of the extraction kinetics for the total EA and PNGs yields are presented in Figure 2. The first 60 min of the kinetics corresponded to the constant extraction rate (CER) period. At this point, the surface area of plant matrix particles was covered with easily accessible solute and mass transfer was dominant [39,41]. The fastest extraction rate was the exp n°2 (40 °C, 30% modifier) and the slowest was exp n°3 (60 °C, 20% modifier). The extraction yield for both conditions at 60 min was 19.04 and 13.96 mg/g respectively. This indicated a 26.68% difference in the yield and validated the influence of lower temperatures for the extraction of the compounds of interest from PPE. As for the exp n°1 (40 °C, 20% modifier) and exp n°4 (60 °C, 30% modifier), for the first 30 min of extraction, both conditions had similar extraction behavior and a yield difference of 7.82%. However, past this time, the extraction rate of exp n°1 was faster and the difference between exp n°1 and n°4 at 60 min was 16.68%. Furthermore, a distinct difference between the experiments carried out at 40 and 60 °C was observed, where at 120 min, the yield was greater at a temperature of 40 °C and was independent from the modifier percentage. The extraction yields for exp n°1 and n°2 around 120 min was of 22.91 and 23.12 mg, respectively, and for exp n°3 and n°4, it was of 20.20 and 20.16, respectively.

These results validated the calculated optimal temperature to extract *Punica granatum* L. pericarp compounds, where the extractions conducted at 40 °C had a superior yield coMPared to higher temperatures (60 °C). In addition, even though exp n°2 had the fastest extraction rate in the first 45 min, after 60 min, exp n°1 had a similar yield with the use of lower amount of modifier. Therefore, the optimal extraction condition selected was exp n°2 with 40 °C and 20% of modifier for 60 min. This extraction condition allowed for the recovery of 18.46 mg/g of compounds of interest from pomegranate pericarp. It was noted that a two-hour extraction resulted in a higher extraction yield of 22.91 mg/g. The second 60 min, however, produced a 24.10% yield increase while consuming twice as many resources. Therefore, 60 min could be sufficient under these conditions to reduce energy and resource consumption, with 40 °C and 20% EtOH:H_2_O 80:20 *v:v*.

### 2.3. Comparison between Supercritical Fluid Extraction (SFE) and Ultrasound-Assisted Extraction (UAE)

The determined optimal extraction conditions in SFE were coMPared to UAE, which is considered a simple and efficient modern extraction technique. The extractions were conducted in coMParable extraction conditions to SFE: plant mass, extraction time, solvent consumption, and composition equivalent to the modifier used.

Two SFEs were conducted in the previously determined optimal conditions for 1g of plant, 60 min, at 40 °C and 20% modifier composed of EtOH:H_2_O 80:20 *v:v* and EtOH:H_2_O 70:30 *v:v*. Indeed, since the percentage of water added to the modifier as an additive demonstrated a significant effect of the yield of compounds of interest from pomegranate pericarp in the experimental design optimization (Table 2), the increase of H_2_O % in the modifier from 20% to 30% was additionally investigated. Furthermore, the UAE was conducted for 60 min using 36 mL of solvent which is in equivalent conditions of time and organic solvent consumption (20% of modifier for 60 min with a total flow of 3 mL/min) with the same ratios of EtOH:H_2_O 80:20 and 70:30 *v:v*.

**Figure 3:**
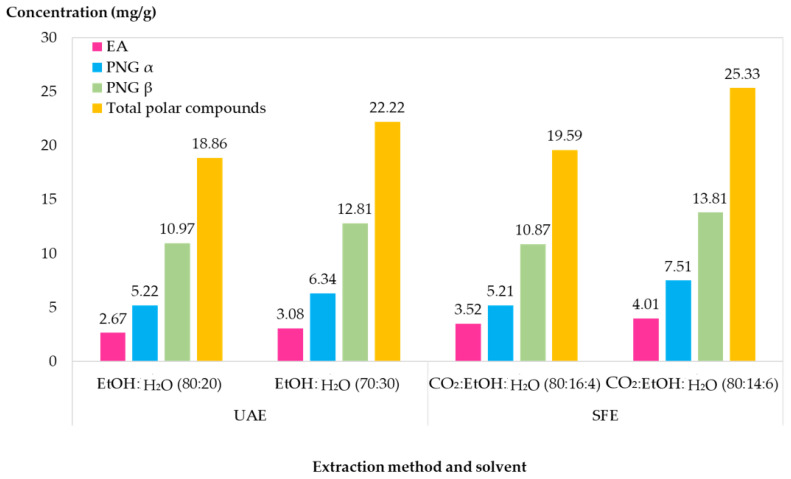
CoMParison between supercritical fluid extraction (SFE) and ultrasound-assisted extraction (UAE) for polar compounds of *Punica granatum* L. pericarp.

The results are presented in Figure 3. For the experiments carried out in UAE when the H_2_O percentage in the extraction solvent increased from 20 to 30%, it resulted in an increase for EA, PNG_α_, and PNG_β_ of 15.35%, 21.45% and 16.77%, respectively.

This indicated that the use of higher H_2_O percentage in the extraction solvent was beneficial for the recovery of pomegranate pericarp polar compounds. Moreover, the experiments performed with SFE exhibited the same behavior as UAE with increase of the of H_2_O percentage. The SC-CO_2_ extraction conducted with a modifier composition of EtOH:H_2_O 70:30 *v:v* when coMPared to 80:20 *v:v*, allowed for a yield increase for EA, PNG_α_, and PNG_β_ of 13.92%, 44.14%, and 27.05% respectively.

Furthermore, when coMParing the extraction yield of both techniques, SFE allowed for an increase of total compounds by 3.87% using EtOH:H_2_O 80:20 *v:v*, and 13.99% using EtOH:H_2_O 70:30 *v:v* coMPared to UAE. This demonstrated that both techniques can extract the compounds of interest from *Punica granatum* L. and similar yields could be recovered. Moreover, even though SFE is mostly known for the extraction of non-polar compounds, it is a coMParable extraction method to UAE for the polar compound’s extraction. Since SFE is a dynamic on-line process, its use can reduce the need for sample or post-extraction preparations (e.g., filtration, centrifugation, and evaporation).

### 2.4. Integrated Process: SFE and Pre-Formulation of Natural Cosmetic Ingredients

During SFE optimization, a difficulty for the solubilization of the dried extracts before analysis in conventional polar and non-polar solvents has been observed. After the screening of many solvent compositions and ratios, DMSO:EtOH:H_2_O 2:1:1 *v:v:v* allowed for a homogenous solubilization of the extracts and was retained for chromatographic analysis. However, this solvent composition is not coMPatible with cosmetic formulations. Thus, in order to bypass the complete evaporation and solubilization of the dried extract to produce cosmetic ingredients, an integrated SFE pre-formulation of extracts process with solvents suitable for cosmetic use was developed. As a result, the extract is maintained in a liquid form and can be used directly for cosmetic formulation.

#### 2.4.1. Dissolution Solvent Screening

An initial screening of cosmetic solvents was carried out to find an adapted composition that allowed a homogenous aspect of the dissolved extracts, avoiding precipitation in time and the use of DMSO which is not coMPatible with cosmetic use. An important point was that the selected solvent does not influence the chromatographic analysis of the PPE to allow the possibility of monitoring the stability of the extracts and the influence of storage duration on compound concentration. Numerous solvents and compositions were selected known for their use in the cosmetic industry. The water percentage was to be limited to 20% in the final composition. This was to reduce any microbiological growth in PPE and to increase the probability of a stable extract, especially since no preservatives were used. A composition of solvent H_2_O 80:20 *v:v* was assessed and, for chromatographic analysis, an additional dilution of the extracts in water was needed to decrease the viscosity of the injected sample.

**Figure 4:**
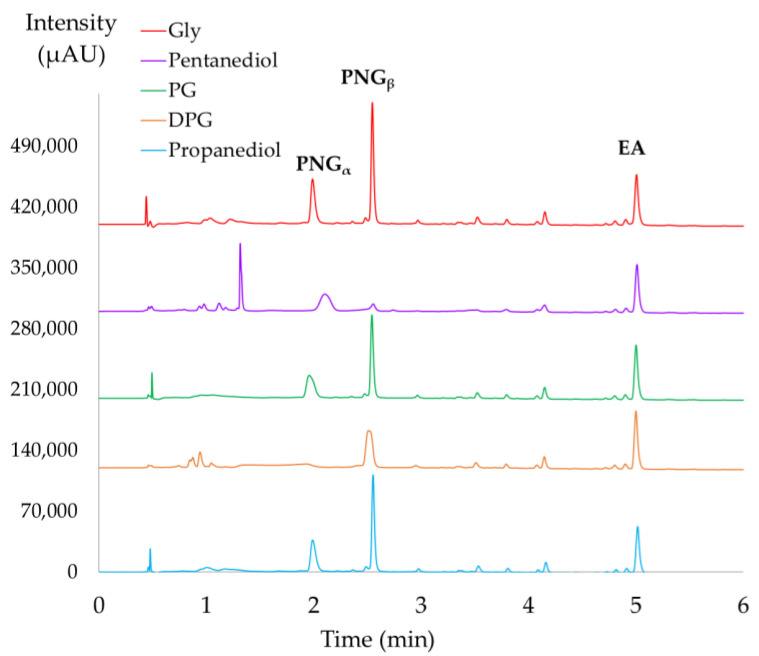
UHPLC-DAD analysis of pomegranate pericarp polar extracts solubilized in cosmetic solvent (composition solvent:H_2_O mixture) at 366 nm. Glycerol (Gly) red, pentanediol purple, propylene glycol (PG) green, di-propylene glycol (DPG) orange, and 1,3-propanediol in blue.

The extracts were showing a homogenous and stable aspect with these cosmetically approved solvents: dipropylene glycol (DPG), propylene glycol (PG), glycerol (Gly), 1,3-propanediol, and pentanediol. The results of UHPLC-DAD analysis of PPE dissolved are presented in Figure 4. Evidently, the EA peak shape was not affected since it was eluted later in the gradient and was independent from the viscosity of the injection solvent. However, the peak shape of PNG_s_ was strongly influenced when using pentanediol and DPG. Moreover, the use of PG influenced the symmetry of PNG_α_. Hence, Gly and propanediol were the only solvents that did not influence the analysis and quantification of PNGs. Lastly, Gly was selected for the stabilization of PPE because of the slight improvement of the peak high, possibly due to enhanced solubility of the studied compounds and the lower price point when compared to propanediol.

#### 2.4.2. Integrated Pre-Formulation of Pomegranate Pericarp SC-CO_2_ Extracts

**Figure 5:**
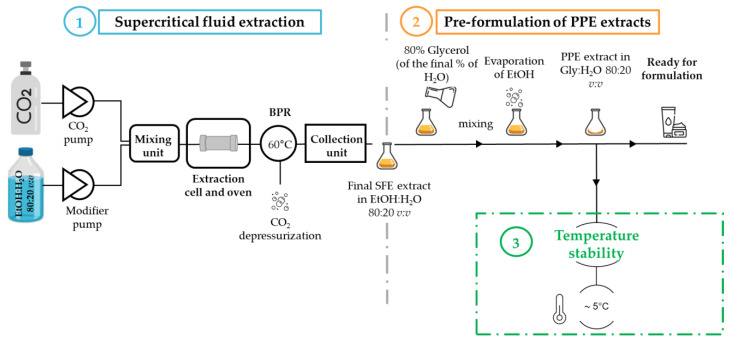
Illustrated scheme of the integrated SFE (step one) and pre-formulation of the final extracts with cosmetic solvents (step two). The stability study evaluation of the pre-formulated extract (step three).

The integrated process for SFE extraction of *Punica granatum* L. pericarp is composed of two main steps, illustrated in Figure 5. The first step is the supercritical fluid extraction of the plant mass using a mixture of SC-CO_2_ and modifier as extraction solvent. As described previously, the optimized extraction conditions (EtOH:H_2_O 80:20 *v:v*) were employed to limit the H_2_O % to 20% of the final extract. After the back-pressure regulator (BPR), the CO_2_ depressurizes and returns to its gaseous state and the final SFE extract is collected in the modifier. The second step included the pre-formulation of the SFE extract in a Gly:H_2_O 80:20 *v:v* this composition allowed the stabilization of the extracts while keeping only 20% of H_2_O, minimizing the influence of water on the stability of the extracts. To achieve this composition, a volume of Gly equal to EtOH was added to the final SFE extract, representing 80% of the final collected volume. Then, the mixtures of EtOH:Gly:H_2_O were mixed manually to achieve a homogenous mixture. The following step included the evaporation of EtOH using nitrogen flow. The evaporation of EtOH first and then adding Gly after to the extract in water was tested. However, the PPE was not fully stable in only water, and this eventually led to its precipitation and later needed centrifugation, adding a unit operation to the post-treatment process. Moreover, the direct addition of glycerol (5%) mixed with ethanol in the modifier during the extraction step was tested and did not achieve a high extraction yield of targeted compounds. Therefore, Gly was added directly to the SFE extract and EtOH was later evaporated. Finally, after this step, the pomegranate extract was solubilized in a Gly:H_2_O 80:20 *v:v* mixture, pre-formulated to be included in cosmetic emulsions of formulas.

#### 2.4.3. Stability Evaluation of the Pre-Formulated Extracts

The pre-formulated PPE were stored at 5 °C. This temperature was chosen since the extracts did not contain any preservatives. Evaluation of the stability was conducted following UHPLC-DAD quantification of compounds concentration over time. Samples were analyzed eight times in triplicate over the course of the 104 days that followed the integrated extraction and pre-formulation process. The data were presented as the concentrations of the compounds of interest in mg/g of biomass and their relative variation percentage was coMPared to the initial values at day one, with a positive variation indicating an increase and a negative a decrease (Table 3). At the final 104th day, the yield of EA increased by 0.27% while that of PNG_α_ and PNG_β_ decreased by 0.06 and 1.97%, respectively, indicating a minimal variability of the compounds of interest. This proved that the *Punica granatum* L. pericarp SFE extracts in Gly:H_2_O 80:20 *v:v* were stable when stored at a low temperature of 5 °C without the use of chemical preservatives.

## 3. Materials and Methods

### 3.1. Plant Material

The plant material for pomegranate (*Punica granatum* L.) consisted of the dried pericarp, milled into fine light orange yellow powder, supplied by PMA 28 (Varize, France). The biomass was stored at room temperature in an airtight container.

### 3.2. Chemical and Reagents

CO_2_ gas was supplied by Air Liquide (Fleury-les-Aubrais, France). HPLC-grade methanol (MeOH), ethanol (EtOH), and dimethyl sulfoxide (DMSO) were used for the mobile phase, extraction, and sample dilution were supplied by VWR (Fontenay-sous-Bois, France). Formic acid (FA), pentanediol, glycerol (Gly), 1,3-propanediol, and diatomaceous earth were obtained from Sigma-Aldrich (Merck, Semoy France). Propylene glycol (PG) and dipropylene glycol (DPG) were provided by Alban Muller (Fontenay-sur-Eure, France). Ultra-pure water was purified by using an Milli-Q^®^ EQ 7000, from Sigma-Aldrich with a resistance > 18 MΩ.cm. Ellagic acid standard used to identify and quantify the target molecules was provided by Extrasynthese (Genay, France).

### 3.3. UHPLC-DAD Analysis

All the extract analyses were performed using a Nexera-LC40 system of Shimadzu Corporation (Kyoto, Japan); this system is equipped with a photodiode array (PDA) detector (SPD-40), a solvent delivery unit (LC-40), an auto-sampler (SIL-40), a column oven (CTO-40), and a system controller (SCL-40). All chromatograms were recorded on LabSolutions LC-UV 5.97 SP1 version (Shimadzu Corporation, Kyoto, Japan).

A Cortecs C18 (100 × 3.0 mm) column coupled to a Cortecs C18 VanGuard Cartridge (90 Å, 5 × 2.1 mm) both packed with 2.7 µm superficially porous particles, from Waters Corporation (Milford, MA, USA) was used for the analysis of all extracts. The column temperature was maintained at 30 °C. The injection volume was 5 µL and the automated sampler was kept at 10 °C. The flow rate was maintained at 1 mL/min. Equilibration time between two injections was 5 min.

**Figure 6:**
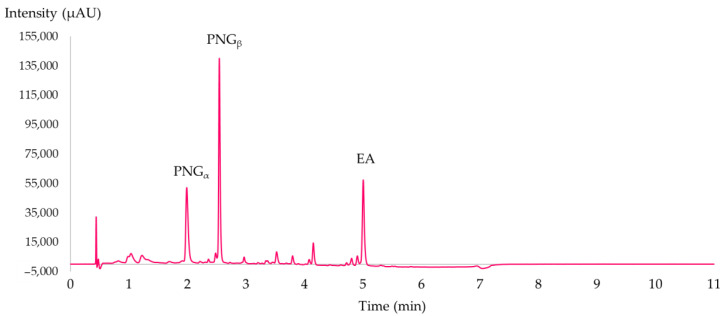
UHPLC-DAD analysis of polar extracts of *Punica granatum* L. pericarp diluted in DMSO:EtOH:H_2_O at 366 nm. PNG α (RT = 1.83 min), PNG β (RT = 2.45 min), and EA (RT = 4.88 min).

The separation Is presented in Figure 6. The total time of each analysis was 12 min, and the mobile phase consisted of a combination of acidified water with 0.1% of FA (solvent A) and MeOH (solvent B).

The elution gradient employed was the following for solvent B (%): 0–0.5 min: 5%; 0.5–6 min: 5–65%; 6–6.1 min: 65–5% and 6.1–11 min: 5%. Detection of the compounds was performed using a PDA detector set at 366 nm (maximal absorbance of EA) and 380 nm (maximal absorbance PNGs).

Quantitative analyses of EA and PNGs were performed by injecting an ellagic acid standard at 11 different concentrations from 0.0005 mg/mL to 0.9 mg/mL. The calibration curve was obtained at 366 nm (y = 10^7^x + 4916.4, R^2^ = 0.9993), and it was used to estimate the concentrations from the peak areas of EA, PNGs (concentrations were quantified as an equivalent to EA; this was possible due to their similar molar absorption coefficient (ε) at their respective maximal absorbance wavelength). In all data presented, the extraction yield was defined as the yield of EA and PNGs (in equivalent to EA), quantified in UHPLC per one gram of plant material (mg/g of biomass). As for the PNGs, both anomers were separated and the identification was performed according their PDA spectra and validated by the literature, where the anomer α was eluted before β in reversed phase LC, with the latter being the major punicalagin [19,27,29].

### 3.4. Supercritical Fluid Extraction (SFE)

A Waters MV-10 ASFE^®^ was used for all the extractions. A 5-mL stainless steel extraction vessel was prepared using 2 g of a plant powder: diatomaceous earth mixture 1:1 *w*:*w*. To filter the extracts and fill the extraction cell entirely, cotton (100%) was placed at the top and bottom of the extraction cell. The extraction duration for the experimental design was of 30 min with a flow rate of 3 mL/min for 2 min at the beginning of each run, in order to reach fast the required pressure. At the end of the 2 min, the flow rate was reduced to 1.5 mL/min for a total of 28 min, this was performed to increase the repeatability of the extraction. The percentage of the modifier was calculated according to the total extraction flow rate. After the DoE optimization, the SFE kinetics were carried out at a of 3 mL/min flow.

The use of different modifier percentages resulted in the variation of the volume of the extracts for each experimental point. We resolved this variation and coMPared the extraction yield of each experimental point. All extracts were evaporated using a nitrogen gas stream, then resolubilized in 4 mL with a mixture of DMSO:EtOH:H_2_O 2:1:1 *v:v:v*. This solvent composition was necessary to solubilize the extracts and obtain a homogenous stable mixture that will not precipitate in time. The extracts were after diluted with water 3 times to decrease the viscosity of the injection solvent, adapt the solvent composition to the elution gradient, and, in consequence, avoid any deformities in the PNGs peak shape.

### 3.5. Ultrasounds Assisted Extraction (UAE)

The UAE was performed using a Branson 3510 (Bransonic^®^ ultrasonic, Semoy, France) bath purchased from Sigma-Aldrich with a power equal to 130W. In total, 1 g of pomegranate pericarp powder was mixed with the extraction solvent in a 50 mL falcon tube.

The extraction solvents consisted of EtOH:H_2_O mixtures in 80:20 and 70:30 ratios with a volume of 36 mL. The biomass and solvent were combined using a vortex at 10,000 rpm for 60 s. They were later sonicated at room temperature for 60 min. Finally, all extracts were centrifuged at 10,000 rpm for 10 min at 25 °C and filtered using a 0.45 µm polyvinylidene difluoride (PVDF) syringe filter from Agilent Technologies (Les Ulis, France) and directly analyzed without evaporation. For UHPLC analysis, they were subsequently diluted with water.

### 3.6. Experimental Design and Statistical Analysis

Ellistat software 6.4 2020/11 version (Annecy, Auvergne-Rhône-Alpes) was used for the experimental design and data analysis. A Box–Behnken design (BBD) from response surface methodology (RSM) was chosen to establish the model and to determine the response pattern (Table 1). It was used to optimize the supercritical fluid extraction of targeted polar compounds from pomegranate pericarp. The three factors used in this study were temperature (X_1_), total modifier percentage (X_2_), and water percentage added as an additive to the modifier (X_3_), with three levels coded (+1) for the highest, (0) middle, and (−1) the lowest level.

The software was also employed for the graphical analysis of the data and the regression model. The analysis of the variance, ANOVA, was used to determine the significance of the independent variables on the response. This was performed using statistical values like Fisher’s test (F value). Significant factors were defined by a *p*-value < 0.05. Correlation coefficients (R^2^) and adjusted correlation coefficients (adj R^2^) were employed to assess the fitness of the design model. To visualize the effect of the variation between the factors, heat-map plots were used. Optimal extraction conditions were determined by solving the regression equation representing the predictive model. To determine the presence of an outlier in the dataset, the Dixon statistical test was applied to the dataset, and the analysis of residues between the experimental and calculated data were evaluated. The dependent response or output (Y) was the sum of the yield of EA and PNGs (mg/g). The experiments were randomized to maximize the effect of the variability in the response. Five replicates at the middle experimental conditions (X_1_ = 0, X_2_ = 0, and X_3_ = 0) of the design were conducted to evaluate the experimental repeatability. The relative standard deviation (RSD) was estimated to confirm the reproducibility of the extraction model. The second-degree polynomial, Equation (2), represents the relationship between the response and the three independent variables (X_1_, X_2_, and X_3_):

Y = β_0_ + β_1_ X_1_ + β_2_ X_2_ + β_3_ X_2 +_ β_11_ X_1_^2^ + β_22_ X_2_^2^ + β_33_ X_3_^2^ (2)

where Y is the predicted response; β_0_ is the regression model constant; β_1_, β_2_, and β_3_ are the coefficients of each variable; β_11_, β_22_, and β_33_ are the quadratic coefficients; and X_1_, X_2_, and X_3_ represent the variables temperature, modifier percentage, and water percentage in modifier, respectively.

The interaction terms X_1_X_2_, X_1_X_3_, and X_2_X_3_ were evaluated during the analysis of the model and their addition decreased the adj R^2^, suggesting overfitting, therefore, justifying their exclusion from the model’s equation.

## 4. Conclusions

In this study, the application of SFE for the extraction of polar compounds of interest from *Punica granatum* L. pericarp was presented. Using an experimental design, the extraction conditions were optimized and the following conditions of 40 °C, 20% modifier of EtOH:H_2_O 80:20 *v:v*, 60 min, 15 MPa, 3mL/min for 1 g of plant, allowed for the recovery of 19.59 mg/g of EA and PNGs, both considered as potent antioxidants. The coMParison between a UAE maceration and SFE in equivalent conditions, particularly the extraction duration, composition, and consumption of solvents demonstrated that SFE is a coMParable extraction technique to UAE and that both methods can offer similar yields in the condition range explored. Additionally, to remedy the issue of precipitation and the difficulty of dissolving dried PPE, a screening of alternative solvents was carried out and a composition of Gly:H_2_O 80:20 *v:v* was selected. This allowed for the development of an integrated SFE and pre-formulation of PPE as a cosmetic ingredient, while at the same time avoiding full evaporation of the extracts. Finally, stability of PPE in Gly:H_2_O 80:20 *v:v* was carried out and a variation of the compounds of interest lower than 2% in three months was achieved at 5 °C.

## Figures and Tables

**Table 1:** Design matrix for the SFE design of experiment (BBD) and responses for polar compounds of interest from *Punica granatum* L. pericarp.

Experiments	Coded Levels			Response (Y): Concentration in mg/g of Biomass after 30 min Extraction			
	X_1_	X_2_	X_3_	Ellagic Acid (EA)	Punicalagine-α (PNG_α_)	Punicalagine-β (PNG_β_)	Total Compounds
	Temperature (°C)	Modifier (%)	H_2_O in Modifier (%)				
1	40 (−1)	10 (−1)	10 (0)	0.000	0.000	0.000	0.000
2	80 (+1)	10 (−1)	10 (0)	0.148	0.002	0.008	0.157
3	40 (−1)	30 (+1)	10 (0)	1.939	0.014	0.232	2.185
4	80 (+1)	30 (+1)	10 (0)	0.838	0.021	0.021	0.880
5	40 (−1)	20 (0)	0 (−1)	0.679	0.015	0.031	0.725
6	80 (+1)	20 (0)	0 (−1)	0.648	0.000	0.003	0.651
7	40 (−1)	20 (0)	20 (+1)	2.104	3.111	6.690	11.905
8	80 (+1)	20 (0)	20 (+1)	2.111	2.456	5.391	9.958
9	60 (0)	10 (−1)	0 (−1)	0.082	0.003	0.009	0.095
10	60 (0)	30 (+1)	0 (−1)	0.932	0.349	0.712	1.994
11	60 (0)	10 (−1)	20 (+1)	0.033	0.006	0.018	0.058
12	60 (0)	30 (+1)	20 (+1)	1.579	0.926	1.969	4.474
13	60 (0)	20 (0)	10 (0)	0.718	0.001	0.003	0.722
14	60 (0)	20 (0)	10 (0)	0.756	0.002	0.006	0.764
15	60 (0)	20 (0)	10 (0)	1.038	0.002	0.006	1.046
16	60 (0)	20 (0)	10 (0)	1.033	0.010	0.023	1.066
17	60 (0)	20 (0)	10 (0)	0.402	0.008	0.017	0.427

**Table 2:** ANOVA for response surface regression model for total compounds of interest (EA and PNGs) yield from *Punica granatum* L. pericarp SFE extracts. (Sig = significant, Nsig = not significant).

Source	DF	F Value	*p*-Value	Contribution (%)	Conclusion
Regression model	6	5.4351	0.0097		Sig
X_1_-Temperature (°C)	1	4.1894	0.0679	11.3	Sig
X_2_-Modifier (%)	1	5.0107	0.0491	13.6	Sig
X_3_-H_2_O in modifier (%)	1	1.8249	0.2065	4.94	NSig
X_1_^2^	1	3.9929	0.0736	10.8	Sig
X_2_^2^	1	3.9910	0.0737	10.8	Sig
X_3_^2^	1	7.9267	0.0183	21.5	Sig
Residuals	10			27.1	
Total	16				
	Degree of freedom				

**Table 3:** Stability evaluation of PPE solubilized in Gly:H_2_O 80:20 mixture. The results are presented in terms of concentration in mg/g of plant and the relative variation percentage (day 104) coMPared to the initial concentration values at day one of the compounds of interest.

EA (mg/g)		PNG_α_ (mg/g)		PNG_β_ (mg/g)	
Day 1	Day 104	Day 1	Day 104	Day 1	Day 104
0.612	0.614 (+0.27%)	0.483	0.483 (−0.06%)	0.783	0.768 (−1.97%)

## Data Availability

Data are contained within the article.
